# Modified Si-Miao Pill for Rheumatoid Arthritis: A Systematic Review and Meta-Analysis

**DOI:** 10.1155/2020/7672152

**Published:** 2020-05-21

**Authors:** Hui Wang, Yao Huang, Pan Shen, Yu Wang, Kai Qin, Ying Huang, Xin Ba, Weiji Lin, Zhe Chen, Shenghao Tu

**Affiliations:** Institute of Integrated Traditional Chinese and Western Medicine, Tongji Hospital, Tongji Medical College, Huazhong University of Science and Technology, Wuhan, Hubei 430030, China

## Abstract

**Objective:**

The aim of this review and meta-analysis was to assess the effects and safety of modified Si-Miao pill (mSMP) in treatment of rheumatoid arthritis.

**Design:**

A systematic literature search was carried out in eight databases from their available dates of inception to April 2020. After screening, fifteen randomized, controlled trials (RCTs) comparing the effects and safety of mSMP in combination with western medicine (including disease-modifying antirheumatic drugs (DMARDs) and nonsteroidal anti-inflammatory drugs (NSAIDs)) in treating rheumatoid arthritis patients were included after screening.

**Results:**

In comparison with DMARDs, or coadministration of DMARDs and NSAIDs, mSMP in combination with western medicine significantly lowered erythrocyte sedimentation rate (mean difference (MD) = -10.61, 95% confidence interval (CI) [−12.19, −9.03]), C-reactive protein (MD = −6.50, 95% CI [−8.43, −4.56]), rheumatoid factors (MD = −17.31, 95% CI [−24.34, −10.27]), swollen joint count (MD = −1.63, 95% CI [−2.29, −0.97]), tender joint count (MD = −1.98, 95% CI [−2.34, −1.62]), and morning stiffness time (MD = −24.37, 95% CI [−29.41, 19.33]) and ameliorated the condition of patients (odds ratio (OR) = 3.69, 95% CI [2.64, 5.14]). Additionally, mSMP in combination with western medicine seemed safer (OR = 0.49, 95% CI [0.30, 0.81]).

**Conclusion:**

The results of the meta-analysis study have shown that mSMP in combination with western medicine therapies appears to be more effective and safer than western medicine alone in the treatment of rheumatoid arthritis including reducing inflammatory markers and adverse events and improving symptoms. Howbeit, more high-grade, large-scale RCTs of mSMP in various countries and regions are still needed.

## 1. Introduction

Rheumatoid arthritis (RA), one of the most common autoimmune diseases, is characterized by symmetrical inflammatory polyarthritis and progressive joint destruction of unknown etiology. It affects approximately 1% of the population at any age, burdening the social economy for its high disability [1, 2]. At present, immunosuppression and anti-inflammatory effects are the main mechanisms of drugs to treat RA. Disease-modifying antirheumatic drugs (DMARDs), including conventional synthetic DMARDs, biological DMARDs, and targeted DMARDs, are thought to be first-line drugs internationally [3]. Nonsteroidal anti-inflammatory drugs (NSAIDs) aim to relieve the pain and inflammation rapidly. However, the various side effects of DMARDs and NSAIDs, such as leukopenia and gastrointestinal, are common and cannot be ignored [4]. Epidemiological studies have found out that patients with RA treated with anti-TNF antibody therapy were at an increased risk of serious infections and a dose-dependent increased risk of malignancies [5]. In addition, huge medical care cost of biological and targeted DMARDs makes it infeasible in many countries, and only 8.3% of patients have received biological DMARDs in China [6]. Furthermore, quite a few people are not sensitive to the current DMARDs. In order to treat RA more effectively and safely with less cost, it is necessary to explore new pharmacologic treatment for RA.

Traditional Chinese medicine (TCM), as a multicomponent and multitarget approach, has been proven to be effective in the treatment of RA in terms of reducing toxicity and increasing the efficacy of mechanisms [7, 8]. Although the use of TCM is common in Southeast Asia, it is rare in other regions. Si-Miao pill (SMP) is primarily composed of Phellodendri Chinensis cortex, atractylodes rhizome, achyranthis bidentatae radix, and Coicis Semen. It has been widely used in the treatment of various arthralgia diseases, especially rheumatoid arthritis in China. According to modern pharmacology, the Si-Miao pill and numerous monomers extracted from it are also invalidated efficaciously in treating RA in vitro and in vivo [9]. The modified Si-Miao pill (mSMP) is derived from the Si-Miao pill, adjusting the composition according to different syndrome types for more effectiveness [10–15]. While mSMP has been applied in RA for a long time, a systematically evidence-based study is vacant. We assessed the effects and safety of mSMP combined with western medicine (DMARDs and NSAIDs) in the treatment of RA systematically by the meta-analysis of randomized controlled trials (RCTs).

## 2. Methods

This study was designed according to the Cochrane Handbook for Systematic Reviews of Interventions and Preferred Reporting Items for Systemic Review and Meta-analyses (PRISMA) guidelines to ensure accuracy and reliability [16]. The review protocol was registered in the PROSPERO database before the start of the review process (CRD42019133738). The PRISMA checklist was presented in Appendix 1 in Supplementary Materials.

### 2.1. Search Strategy

Only Chinese and English articles were concerned. We conducted a systematic literature search including eight Chinese and foreign databases to ascertain trials including CNKI Databases, Wan Fang Database, Chinese Biomedical Literature database (CBM), PubMed, EMBASE, the Cochrane Library, Clinical Trails, and Web of Science. All of the databases were searched from their available dates of inception to April 2020. Search strategies were combined as follows. For the English databases, free text terms ((rheumatoid arthritis) OR (rheumatism)) AND ((si miao) OR (four subtleties)) were applied. For the Chinese databases, the terms were (si miao AND (lei feng shi guan jie yan OR lei feng shi guan jie yan (RA in Chinese)) NOT si miao yong and NOT si miao xiao bi).

### 2.2. Selection Criteria

We adopted the following criteria: (1) studies used mSMP in combination treatment with western medicine (including DMARDs and NSAIDs); (2) all partaken patients who were diagnosed with RA according to the authoritative diagnostic criterion of RA such as the 1987 or 2010 American College of Rheumatology (ACR)/European League Against Rheumatism Criteria; and (3) RCTs.

### 2.3. Exclusion Criteria

Exclusion criteria were as follows: (1) studies using mSMP alone, si miao yong an, and si miao xiao bi decoction were excluded; (2) case reports, reviews, and animal experiments were excluded; (3) diagnosis criteria are unclear; (4) studies without regulatory outcomes or studies in which the evaluation of curative effect is not standard were excluded; (5) articles that have no available full text were excluded.

### 2.4. Types of Outcome Measures

The primary outcomes were the main indicators correlating with disease activity including sedimentation rate (ESR), C-reactive protein (CRP), rheumatoid factors (RF), swollen joint count, and tender joint count. The secondary outcomes were some clinical symptoms reflecting disease activity consisting of effective rate, morning stiffness times, and adverse events (AEs). The decrease of ESR, CRP, RF, morning stiffness times, swollen joint count, and tender joint count and the increase of effective rate could reflect the therapeutic effects (TEs). AEs included abnormal liver function, hyperleukocytosis, acratia, erythema or itch of the skin, dental ulcer, and gastrointestinal discomfort.

### 2.5. Data Extraction and Management

The data were extracted by two independent reviewers (HW and YAH). Any discrepancy was resolved by consensus or judged by the corresponding author (SHT and ZC). All relevant data including characteristics of trails, the first author, year of publication, baseline characteristics of patients, number of patients, duration of the study, intervention methods, and outcome measures were extracted and entered into a template table. We assessed the included studies' quality on the basis of Cochrane Collaboration's risk of bias tool. There were three scores for each item, low risk, unclear, and high risk, according to following criteria: (1) random sequence generation, (2) allocation concealment, (3) blinding of participants and personnel, (4) incomplete outcome data, (5) selective reporting, and (6) other biases.

### 2.6. Statistical Synthesis and Analysis

Review Manager 5.3 and Stata 12.0 were used to calculate the differences of effects and safety of mSMP in treating RA between the mSMP/experimental groups (mSMP treatment in combination with DMARDs that were combined or were not combined with NSAIDs) and the control groups (DMARDs that were combined or were not combined with NSAIDs). We applied the odds ratio (OR) and 95% confidence interval (CI) to appraise dichotomous data and mean difference (MD) to continuous data. Heterogeneity was evaluated according to the chi-square test and the Higgins *I*^*2*^ test. The fixed-effects model was applied when statistical heterogeneity was low that was *I*^*2*^ ≤ 50% or Chi^2^ test *P* < 0.10; otherwise, a random-effects model was employed. The subgroup analysis was performed to eliminate heterogeneity, and sensitivity analysis was adopted to probe the source of heterogeneity. If the data provided by the included studies were not appropriate for performing a meta-analysis, the study data were presented in narrative form. Publication bias was detected by Egger's regression asymmetry test.

## 3. Results

### 3.1. Study Selection and the Basic Documents

**Figure 1:**
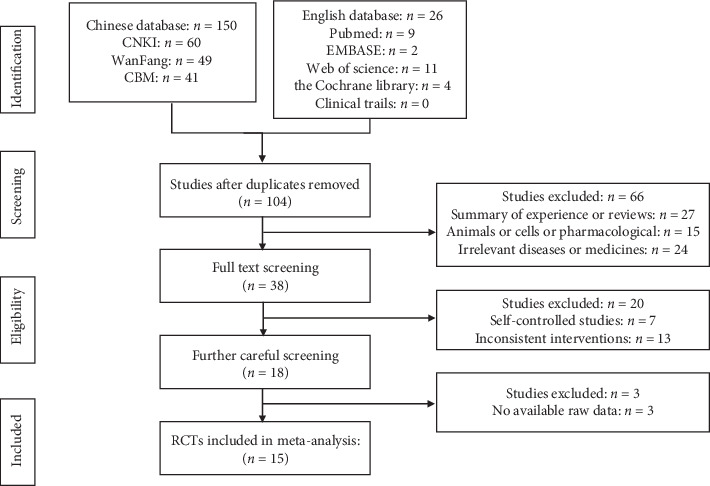
Study selection flow chart.

The flow chart of the study selection was given in Figure 1. In the primary screening, we retrieved 176 articles. After removing duplicates, titles and abstracts of 104 studies were screened. 27 records were excluded because they were a summary of experience or reviews (*n* = 27), animals or cell experiments (*n* = 15), or irrelevant diseases or medicines (*n* = 24). After the full-text reading of the resulting 38 studies, 20 records were excluded, seven of which were due to self-controlled studies and thirteen of which were owning to inconsistent interventions and four of which were due to no available raw data. Eventually, 15 RCTs were enrolled in the meta-analysis, including 1349 participants in total [17–31]. The characteristics of the studies were exhibited in Table 1. They were all carried out in China and published between 2007 and 2019 in Chinese. All of them were conducted as single-center trials. There was no statistically significant difference in gender and age between treatment groups and the control groups in the literature, and the course of the disease as well. The duration of intervention in the included RCTs ranged from 30 to 90 days. The dosage of mSMP was twice a day and the dosage of DMARDs or NSAIDs was the same between experiment (mSMP) and control groups. DMARDs in these RCTs included Methopterin (MTX), Leflunomide (LEF), Salazosulfapyridine (SASP), and Hydroxychloroquine (HCQ), while SAIDs included Diclofenac sodium, meloxicam, Voltaren, and Loxoprofen. Fourteen RCTs [17–23, 25–31] had definite TCM syndrome of the patients according to the criteria of diagnosis and therapeutic effect of diseases and syndromes in TCM, of which eleven [17, 19, 21–23, 25–29, 31] were dampness-heat, and three [18, 20, 30] were wind and dampness-heat. The formulations and compositions of mSMP were listed in Table 2. The decoction was used in thirteen articles [18–30] and the pill was used in two articles [17, 31]. The dosage of Phellodendri Chinensis cortex is from 10 to 15 g and that of Coicis Semen is from 15 to 30 g; the dosage of achyranthis bidentatae radix is from 15 to 30 g and that of atractylodes rhizome is from 10 to 15 g. Four articles [17, 28, 29, 31]adopted the original formula of SMP while other studies added herbs based on SMP.

### 3.2. Quality Assessment of Included Studies

**Figure 2:**
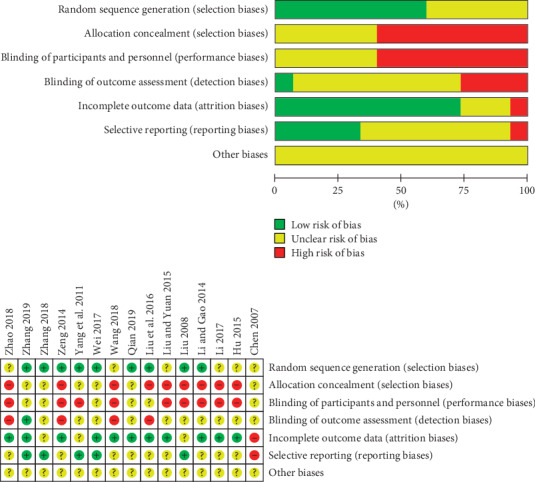
Risk of bias graph.

Most of the included RCTs were of poor quality according to the Cochrane Collaboration's risk of bias tool criteria shown in Figure 2. Eight [17–19, 21, 22, 25, 28, 31] of the included studies indicated random sequence generation, two [19, 21] of which are based on treatment order, one [18] of which was based on sortition randomization method, and the remaining five [17, 22, 25, 28, 31] were based on the table of random numbers. None of the articles mentioned allocation concealment or blind method, as well as intentional analysis. All patients completed the experiments, and no one lost the interview or dropped out. None of the trials reported other biases.

### 3.3. Publication Bias

Egger's publication bias test showed that there were negligible publication biases for four outcomes in terms of RF (*P*=0.309), morning stiffness time (*P*=0.065), swollen joint count (*P*=0.092), and tender joint count (*P*=0.734) while there were significant publication biases for four outcomes in terms of ESR (*P*=0.001), CRP (*P*=0.014), effective rate (*P*=0.04), and AEs (*P*=0.032) for the evaluation rules that studies with *P* values over 0.05 in Egger's test were deemed low heterogeneity. The effects of lowering ESR and reducing need further exploration, as presented in Appendix 2 in Supplementary Materials.

### 3.4. TEs of mSMP

#### 3.4.1. ESR (mm/h), CRP (mg/L), RF (IU/mL), Swollen Joint Count, and Tender Joint Count

**Figure 3:**
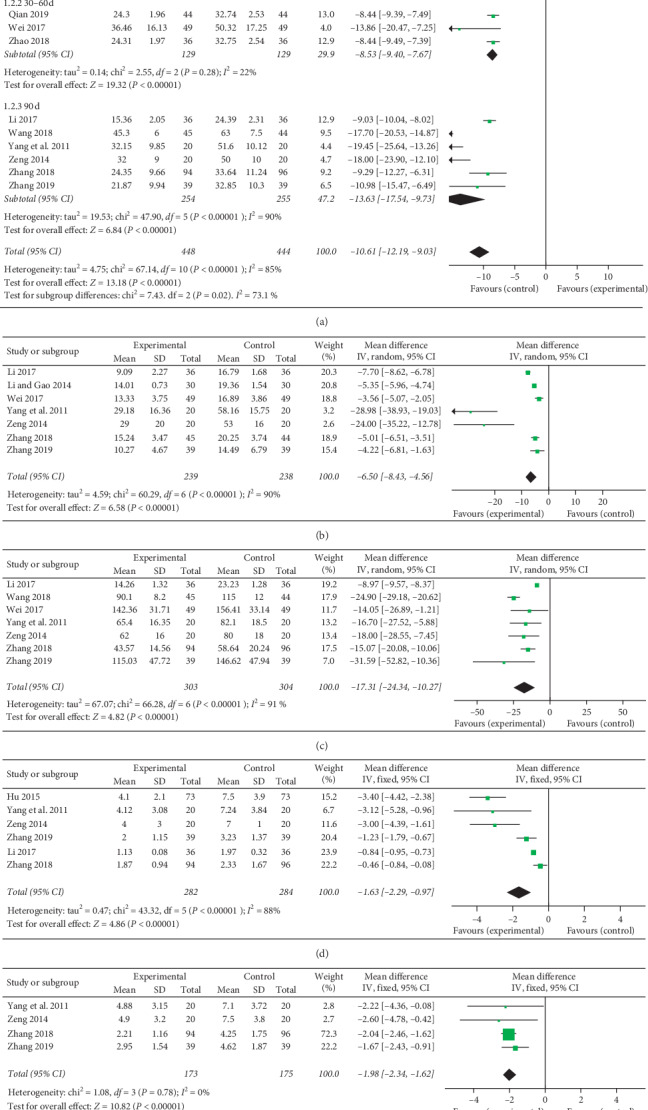
Forest plots for the comparison of ESR (a), CRP (b), RF (c), swollen joint count (d), and tender joint count (e) of mSMP treatment in combination with western drugs (DMARDs and NSAIDs) (experimental) and western drugs (control) only. Note: ESR: erythrocyte sedimentation rate; CRP: C-reactive protein; RF: rheumatoid factors; AEs: adverse events; mSMP: modified Si-Miao pill; DMARDs: disease-modifying antirheumatic drugs; NSAIDs: nonsteroidal anti-inflammatory drugs (NSAIDs).

ESR was reported in eleven trials (involving 892 patients) [17–19, 21, 22, 24, 25, 27, 28, 30, 31]. The number of trial participants ranged from 40 to 190, with the trial duration varying from 30 days to 90 days. The mSMP groups were superior to the control groups regarding decreasing the ESR (MD = −10.61, 95% CI [−12.19, −9.03]). In order to reduce the high heterogeneity (*I*^*2*^ = 85%, *P* < 0.00001) and explore the effect of treatment time, we stratified studies based on trial duration (30 d, 30–60 d, 90 d). The analysis of the subgroups showed that trial duration could be one of the potential sources of heterogeneity (*I*^*2*^ = 73.1%, *P*=0.02). Additionally, the treatment effect of mSMP seemed to be time-dependent (30 d: MD −7.68, CI [−9.45, −5.92]; 30–60 d: MD −8.53, CI [−9.40, −7.67]; 90 d: MD −13.63, CI [−17.54, −9.73]), as illustrated in Figure 3(a).

CRP was determined in seven trials (involving 477 patients) [17, 19, 21, 25, 27, 28, 31]. The number of trial participants ranged from 40 to 98, with the trial duration varying from 30 days to 90 days. The experimental groups were superior to the control groups regarding reducing the CRP CRP (MD = -6.50, 95% CI [−8.43, −4.56]). There was statistical heterogeneity between the studies based on the random-effects model (*I*^*2*^ = 90%, *P* < 0.00001), as illustrated in Figure 3(b).

RF was determined in seven trials (involving 607 patients) [17, 21, 24, 25, 27, 28, 31]. The number of trial participants ranged from 40 to 190, with a 90-day trial duration. As illustrated in Figure 3(c), there was statistical heterogeneity between the studies based on the random-effects model (*I*^*2*^ = 91%, *P* < 0.00001). The experimental groups were superior to the control groups regarding reducing the RF (MD = −17.31, 95% CI [−24.34, −10.27]).

The swollen joint count was determined in six trials (involving 566 patients) [17, 25, 27–29, 31]. The number of trial participants ranged from 40 to 190, with a 90-day trial duration. As illustrated in Figure 3(d), there was statistical heterogeneity between the studies based on the random-effects model (*I*^*2*^ = 88%, *P* < 0.00001). The experimental groups were superior to the control groups regarding reducing the swollen joint count (MD = −1.63, 95% CI [−2.29, −0.97]).

The tender joint count was determined in four trials (involving 348 patients) [17, 25, 28, 31]. The number of trial participants ranged from 40 to 190, with a 90-day trial duration. As illustrated in Figure 3(e), there was no statistical heterogeneity between the studies based on the fixed-effects model (*I*^*2*^ = 0%, *P*=0.78). The experimental groups were superior to the control groups regarding reducing the tender joint count (MD = −1.98, 95% CI [−2.34, −1.62]).

#### 3.4.2. Effective Rate and Morning Stiffness Time (min)

**Figure 4:**
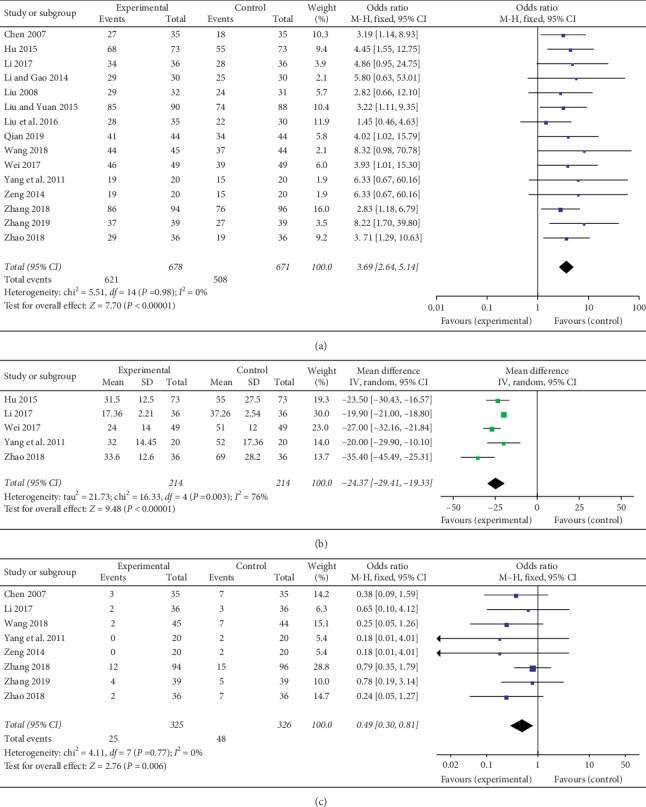
Forest plots for the comparison of effective rate (a), morning stiffness time (b), and adverse events (c) of mSMP treatment in combination with western drugs (DMARDs and NSAIDs) (experimental) and western drugs only. mSMP: modified Si-Miao pill; DMARDs: disease-modifying antirheumatic drugs; NSAIDs: nonsteroidal anti-inflammatory drugs (NSAIDs).

Concerning mSMP treatment in combination with DMARDs and NSAIDs (or no NSAIDs), fifteen trials (involving 1349 patients) compared the effective rate of mSMP groups with control groups [17–31]. Patients reaching ACR20 or efficient according to the criteria for diagnosis and efficacy of TCM diseases and syndromes were considered as effective. The trial duration varied from 30 days to 90 days. There was no statistical heterogeneity between the studies based on the fixed-effects model (*I*^*2*^ = 0%, *P*=0.98). The experimental groups were more effective in improving the condition (OR = 3.69, 95% CI [2.64, 5.14]), as illustrated in Figure 4(a).

Morning stiffness time was determined in five trials (involving 428 patients) [21, 27–30]. The number of trial participants ranged from 40 to 146, with the trial duration varying from 42 days to 90 days. As illustrated in Figure 4(b), there was statistical heterogeneity between the studies based on the random-effects model (*I*^*2*^ = 76%, *P* < 0.00001). The experimental groups were superior to the control groups regarding the reduction of the morning stiffness time (MD = −24.37, 95% CI [−29.41, 19.33]).

### 3.5. AEs

AEs were reported in eight trials (involving 651 patients) [17, 23–25, 27, 28, 30, 31]. As illustrated in Table 3, the number of cases ranged from 0 to 18. Gastrointestinal discomfort was the most common adverse reaction, of which 18 cases were reported in the control groups and 10 cases in the experimental groups (mSMP + western medicine) according to Chen, Li, Qian, Zeng, Zhao, and Zhang's studies. There were six cases of erythema or itch of skin reported in control groups (western medicine) and only two cases in experimental groups in Zhao's study. Three trials reported hyperleukocytosis with a total of 3 cases in the control groups and no case in the experimental groups, the same as a dental ulcer. Two cases of abnormal liver function were reported in the control groups and only one case occurred in the experimental groups, as illustrated in Figure 4(c). There was no statistical heterogeneity between the studies based on the fixed-effects model (*I*^*2*^ = 0%, *P*=0.77). Compared to the control groups, the mSMP groups could reduce the side effects apparently (OR = 0.49, 95% CI [0.30, 0.81]).

### 3.6. Sensitivity Analysis

In the sensitivity analysis, no significant changes of heterogeneity related to ESR, CRP, and swollen joint count were observed. In terms of RF and morning stiffness time, the study by Li [27]was most likely to be the sources of heterogeneity study. When it was omitted, the change of heterogeneity was significant (RF: *I*^*2*^ reducing from 91% to 55%, morning stiffness time: *I*^*2*^ reducing from 76% to 44%), and the general conclusion remained the same, as presented in Appendix 3 in Supplementary Materials.

## 4. Discussion

Although mSMP has been applied in treating RA for several years and there were massive clinical reports and clinical trials in China, it is the first time to evaluate the efficacy and safety of mSMP in the treatment of RA by systematic reviews and meta-analyses. Biomarkers play an important role in guiding the clinical trials and therapies of RA. ESR and CRP are the most common experimental indicators reflecting the inflammatory activity of RA, which is useful to evaluate the condition and prognosis. RF is an antibody correlated with RA and predicts bone erosion and severe disease progression [32]. It generally does not change much for a short time, but its change can explain the disease to a certain extent. ACR20 and criteria for diagnosis and efficacy of TCM diseases and syndromes are the most common and authoritative standards to evaluate the efficacy of drug therapy. Swollen joint count, tender joint count, and morning stiffness time are the characteristic manifestations of RA which played a great role in assessing the disease activity. According to the 2018 Chinese guideline for diagnosis and treatment of rheumatoid arthritis, ESR, CRP, RF, anti-cyclic citrulline antibody, swollen joint count, and tender joint count should be taken into account in the treatment of RA. Since there were few studies on the measurement of anti-cyclic citrulline antibody, ESR, CRP, RF, swollen joint count, and tender joint count were regarded as primary outcomes. This statistical analysis revealed that mSMP in combination with western medicine seemed to be more effective and significant in reducing the levels of ESR, CRP, and RF, ameliorating morning stiffness time, swollen joint count, tender joint count, and the incidence of AEs incidence. Due to the limited studies included, we could not conduct subgroups' analysis in terms of dosage, treatment durations, the difference of control drugs to remove the high heterogeneity of CRP, and swollen joint count. We speculated on several possible reasons, such as patients with different disease activities, different regions, and not enough samples, which needs more and better quality clinical trials to explore. Furthermore, although the included article did not analyze whether the adverse events were caused by mSMP or western medicine in experimental groups, mSMP combined with western medicine had fewer adverse events than western medicine.

RA belongs to “bi” in traditional Chinese medicine, while a large number of active RAs vest in “dampness-heat of bi” [7]. TCM syndrome of most of the patients included in the RCTs was dampness-heat. SMP was written by Zhang Bingcheng in the Qing Dynasty famous for the definite effect of clearing heat and dampness, reducing swelling, and relieving pain [33]. The prescription medicine mainly includes cortex Phellodendri, rhizoma atractylodes, radix achyranthis bidentatae, and Semen Coicis, modified according to the individual symptom for being a more-targeted treatment, which is a mere coincidence with precision medical treatment. On the other hand, the difference in composition and dosage of the prescriptions is also a disadvantage of standard evaluation.

mSMP in the treatment of RA has also been confirmed according to modern pharmacology in vivo and in vitro. SMP was found to significantly inhibit the expression of IL-1*β*, IL-6, and TNF-*α* in adjuvant arthritis (AA) rats [34]. mSMP extract inhibited the release of inflammatory mediators, like NO and TNF-*α*, via the suppression of ERK and NF-*κ*B-dependent pathways from lipopolysaccharide-stimulated mouse macrophages [14]. Wang found that SMP could downregulate the expression of VEGF in AA rats, thus inhibiting the formation of synovia pannus [35]. In addition, cortex Phellodendri, rhizoma atractylodes, and Semen Coicis had obvious anti-inflammatory and analgesic effects. Total saponins of *Achyranthes bidentata* could attenuate the acute inflammatory reaction and regulate immunity [36, 37]. Moreover, SMP and one of the main components, berberine, were confirmed to regulate the blood lipids, increase the level of high-density lipoprotein, which is thought to improve rheumatism and benefit the cardio-cerebrovascular disease in terms of the common view that patients with rheumatoid arthritis are at increased risk of cardiovascular disease [38–40]. In condition, mSMP may lower CVD occurrences by lipid regulation and well work in hormonal-dependent RA [41]. One trial reported that mSMP could lower the recurrence rate during the one-year follow-up period.

There were also a lot of shortcomings in this study. First, all the included RCTs were conducted in China and might cause selection bias. Most of the included RCTs were of poor quality according to the Cochrane Collaboration's risk of bias tool criteria for a missing message of allocation concealment, blind method, and intentional analysis. Second, the form, compositions, dosages, and treatment duration of mSMP were complex and changeable, which might undermine the credibility. Furthermore, the studies included in the systematic review were few in number and there was no unified standard for disease evaluation. Finally, the lack of relevant grey literature may lead to publication bias.

## 5. Conclusions

Overall, mSMP combined with western medicine was more effective and safer than western medicine in the treatment of RA. mSMP may play the pharmacological action by anti-inflammation, regulating immunity, analgesia, and lipid regulation. In consideration of the low quality, single area of the given trials, and variable interventions, more large randomized controlled, double-blind, multicenter clinical trials with good methodological quality are needed to recommend mSMP as an alternative remedy for RA.

## Figures and Tables

**Table 1:** Clinical and demographic characteristics of patients with rheumatoid arthritis.

Study (ref)	Number of participants experimental/control	Age (years) experimental/control	Intervention		Duration (days)	Outcomes	TCM syndrome
			Experimental	Control			
Chen [23]	35/35	50/50	mSMP + MTX 7.5 + LEF 10	MTX 7.5 + LEF 10	90	Effective rate AEs	Dampness-heat
Hu [29]	73/73	43.5 ± 5.0/43.0 ± 5.5	mSMP + MTX 7.5 + Voltaren 75	MTX 7.5 + Voltaren 75	90	Effective rate ESR RF MST SJT AEs	Dampness-heat
Li [27]	36/36	47.00 ± 2.35/48.00 ± 2.19	mSMP + MTX 10 + Loxoprofen sodium 60	MTX 10 + Loxoprofen sodium 60	90	Effective rate ESR CRP RF MST SJT AEs	Dampness-heat
Li and Gao [19]	30/30	52.16 ± 10.24/54.02 ± 14.76	mSMP + MTX 10 + Voltaren 75	MTX 10 + Voltaren 75	30	Effective rate ESR CRP	Dampness-heat
Liu [22]	32/31	44.5 ± 11.6/43.6 ± 13	mSMP + MTX 5-10 + LEF 10	MTX 5-10 + LEF 10	30	Effective rate	Wind and dampness-heat
Liu and Yuan [26]	90/88	42.18 ±4.76/42.18 ±4.76	mSMP + MTX 15 + SASP 500	MTX 15 + SASP 500	90	Effective rate	Dampness-heat
Liu [22]	35/30	46.89 ±9.15/45.40 ± 8.63	mSMP + LEF 10	LEF 10	30	Effective rate ESR	Dampness-heat
Qian [18]	44/44	44.7 ± 3.9/45.6 ± 3.7	mSMP + MTX 7.5 + Meloxicam 7.5	MTX 7.5 + Meloxicam 7.5	60	Effective rate ESR	Wind and dampness-heat
Wang [24]	45/44	50.1 ± 4.7/48.7 ± 4.6	mSMP + MTX 10	MTX 10	90	Effective rate ESR RF AEs	Unclear
Wei [21]	49/49	42.6 ± 5.7/41.3 ± 5.7	mSMP + MTX 15 + Meloxicam 7.5	MTX 15 + Meloxicam 7.5	42	Effective rate ESR CRP RF MST	Dampness-heat
Yang [28]	20/20	43/41	mSMP + MTX 7.5 + Voltaren 75	MTX 7.5 + Voltaren 75	90	Effective rate ESR CRP RF MST SJT TJC AEs	Dampness-heat
Zeng [17]	20/20	43/41	mSMP + MTX 7.5 + HCQ 0.2	MTX 7.5 + HCQ 0.2	90	Effective rate ESR CRP RF SJT TJC AEs	Dampness-heat
Zhang [31]	94/96	34.54 ± 12.51/35.33 ± 12.52	mSMP + MTX 10 + Loxoprofen sodium 60	MTX 10 + Loxoprofen sodium 60	90	Effective rate ESR CRP RF SJT TJC AEs	Dampness-heat
Zhang [25]	39/39	47.59 ± 10.18/47.95 ± 11.94	mSMP + MTX 7.5-15 + Diclofenac sodium 75	MTX 7.5-15 + Diclofenac sodium 75	90	Effective rate ESR CRP RF SJT TJC AEs	Dampness-heat
Zhao [30]	36/36	47.9 ± 2.4/47.2 ± 2.3	mSMP + MTX 10 + LEF 10	MTX 10 + LEF 10	60	Effective rate ESR MST AEs	Wind and dampness-heat

Note: mSMP: modified Si-Miao pill; TCM: traditional Chinese medicine; MTX : Methopterin mg/week; LEF: Leflunomide mg, bid; Voltaren: mg/day; Loxoprofen sodium: mg/day; Meloxicam: mg/day; SASP: Salazosulfapyridine, mg, bid; HCQ: hydroxychloroquine g/day; Diclofenac sodium: mg/day; AEs: adverse events; ESR: erythrocyte sedimentation rate; CRP: C-reactive protein; RF: rheumatoid factors; MST: morning stiffness time; SJC: swollen joint count; TJC: tender joint count.

**Table 2:** The components of mSMP.

Studies	Formulations	Components of mSMP
Chen [23]	Decoction	Atractylodis rhizome, achyranthis bidentatae radix, Coicis Semen, atractylodes macrocephalae rhizome, alismatis rhizome, sinomenii caulis, piperis kadsurae caulis, dioscoreae nipponicae rhizome, lonicerae japonicae flos, lonicerae japonicae flos, glycyrrhizae radix et rhizome
Hu [29]	Decoction	Phellodendri chinensis cortex, atractylodes rhizome, achyranthis bidentatae radix, Coicis Semen
Li [27]	Decoction	Phellodendri chinensis cortex, atractylodes rhizome, achyranthis bidentatae radix, coicis semen, lonicerae japonicae caulis, smilacis glabbrae rhizome, angelicae sinensis radix, paeoniaeradix rubra, salviae miltiorrhizae radix et rhizome, plantaginis semen, alismatis rhizome, saposhnikoviae radix, astragali radix, gleditsiae apina, glycyrrhizae radix et rhizome
Li and Gao [19]	Decoction	Phellodendri Chinensis cortex, atractylodes rhizome, achyranthis bidentatae radix, Coicis Semen, piperis kadsurae caulis, sinomenii caulis, atractylodes macrocephalae rhizome, smilacis glabbrae rhizome
Liu [22]	Decoction	Phellodendri chinensis cortex, atractylodes rhizome, achyranthis bidentatae radix, Coicis Semen, gentianae macrophyllae radix,
Liu and Yuan [26]	Decoction	Phellodendri chinensis cortex, atractylodes rhizome, achyranthis bidentatae radix, Coicis Semen, sinomenii caulis, glycyrrhizae radix et rhizome
Liu [22]	Decoction	Phellodendri Chinensis cortex, atractylodes rhizome, achyranthis bidentatae radix, Coicis Semen, forsythia fructus, clematidis radix et rhizome, paeoniae radix alba, lysimachiae herba, saposhnikoviae radix, stephaniae tetrandrae radix, lonicerae japonicae caulis, hedyotis diffusa, mori ramulus, violae herba, pheretima, pheretima, glycyrrhizae radix et rhizome
Qian [18]	Decoction	Phellodendri Chinensis cortex, Coicis Semen, stephaniae tetrandrae radix, stephaniae tetrandrae radix, pheretima, lysimachiae herba, violae herba, lonicerae japonicae caulis, mori ramulus, paeoniae radix alba, clematidis radix et rhizoma
Wang [24]	Decoction	Atractylodis macrocephalae, corydalis rhizome, sinomenii caulis, piperis kadsurae caulis, dioscoreae nipponicae rhizome, alismatis rhizome, lonicerae japonicae caulis, lonicerae japonicae flos, trachelospermi caulis et folium, glycyrrhizae radix et rhizome
Wei [21]	Decoction	Phellodendri chinensis cortex, atractylodes rhizome, achyranthis bidentatae radix, Coicis Semen, mori ramulus, chaenomelis fructus, spatholobi caulis, trachelospermi caulis et folium, glycyrrhizae radix et rhizome,
Yang [28]	Decoction	Phellodendri Chinensis cortex, atractylodes rhizome, achyranthis bidentatae radix, Coicis Semen
Zeng [17]	Pill	Phellodendri Chinensis cortex, atractylodes rhizome, achyranthis bidentatae radix, Coicis Semen
Zhang [31]	Pill	Phellodendri Chinensis cortex, atractylodes rhizome, achyranthis bidentatae radix, Coicis Semen
Zhang [25]	Decoction	Phellodendri Chinensis cortex, atractylodes rhizome, achyranthis bidentatae radix, Coicis Semen, semen persicae, rhizoma pinelliae, paeoniae radix rubra, angelicae sinensis radix, bombyx batryticatus, pericarpium citri reticulatae, paeoniae radix alba, radix glycyrrhizae preparata
Zhao [30]	Decoction	Phellodendri Chinensis cortex, atractylodes rhizome, achyranthis bidentatae radix, Coicis Semen, forsythia fructus, clematidis radix et rhizome, paeoniae radix alba, lysimachiae herba, saposhnikoviae radix, stephaniae tetrandrae radix, lonicerae japonicae caulis, hedyotis diffusa, mori ramulus, violae herba, pheretima, pheretima, glycyrrhizae radix et rhizome

**Table 3:** The adverse events about all included RCTs.

Adverse events	Experimental (mSMP + western medicine)		Control (western medicine)	
	*N*	Total	*N* total	Total
Abnormal liver function [27]	1	26	2 36	36
Hyperleukocytosis [18, 23, 30]	0	115	3 115	71
Acratia ([28])	0	20	2 20	20
Gastrointestinal discomfort [18, 23, 25, 27, 30]	10	190	18 190	107
Erythema or itch of skin [18, 30]	2	80	6 80	36
Dental ulcer [17]	0	20	2 20	20

Note: RCTs: randomized, controlled trials; mSMP: modified Si-Miao pill.

## Supplementary Materials

Supplementary Materials Appendix 1: Checklist. Appendix 2: Figure 1: Egger's publication bias in the included trails. A: erythrocyte sedimentation; B: C-reactive protein; C: rheumatoid factors; D: effective rate; E: morning stiffness time; F: swollen joint count; G: tender joint count; H: adverse events. Appendix 3: Figure 2: Sensitivity analysis of ESR (A), CRP (B), RF (C), morning stiffness time (D), and swollen joint count (E). Note: ESR: erythrocyte sedimentation rate; CRP: C-reactive protein; RF: rheumatoid factors.
